# Behavioral Phenotype in Heterozygous DAT Rats: Transgenerational Transmission of Maternal Impact and the Role of Genetic Asset

**DOI:** 10.3390/brainsci12040469

**Published:** 2022-03-31

**Authors:** Greta Manoni, Concetto Puzzo, Antonella Gigantesco, Walter Adriani

**Affiliations:** 1 Faculty of Psychology, Università Telematica Internazionale Uninettuno, 00100 Rome, Italy; g.manoni@students.uninettunouniversity.net (G.M.); concetto.puzzo@alice.it (C.P.); 2 Istituto Superiore di Sanità, Center of Behavioural Science and Mental Health, 00100 Rome, Italy; antonella.gigantesco@iss.it

**Keywords:** intergenerational transmission, epigenotype, circadian cycle, asset, style of care

## Abstract

Dopamine transporter (DAT) is involved in dopamine (DA) reuptake in presynaptic terminals. Deletion of DAT results in a hyperdopaminergic KO-rat phenotype. To conduct our studies in heterozygous DAT rats, several pedigree lines were created, with known derivation of the allele (i.e., maternal or paternal). Our purpose was to elucidate the role of parental origin rather than maternal care, assessing if maternal maltreatments generated sequelae in female offspring. In the first experiment, female rats and their pups were observed during postnatal lactation. Control dams were WT and heterozygous ones were MAT (but K-MAT, with previous experience of early maltreatment by their KO adoptive dams). WT dams were highly attracted to their offspring (predictably, they spent a lot of time licking their pups); in contrast, K-MAT dams showed strangely comparable levels of caring for their pups and exploring the environment. Subsequently, peculiar features of the circadian cycle were found in adolescent rats with different epigenotypes (WT, MUX = offspring of MAT father, MIK = offspring of K-MAT dam). The MIK epigenotype produced locomotor hyperactivity also during resting hours, well above typical values. The MUX epigenotype, on the other hand, was less active and presented a depression-like profile. This study is unique: maltreatment was generated in a spontaneous way from a DAT-KO mother to offspring. We highlight how future studies will address separate contributions by genotype and upbringing. In conclusion, paternal-allele asset generates sequelae diametrically opposed to the inheritance of early maternal trauma.

## 1. Introduction

Dopamine (DA) is the neurotransmitter commonly associated with gratification: it is released within the cortico-limbic and cortico-striatal circuits to gratify us for the positive results of our actions and choices. Such release motivates us to replicate them numerous times until they become rituals, hence creating habits or even addictions. In fact, DA plays an important role both in the implicit memory of behavior and in the motivation of voluntary movement. Instead, the dopamine transporter (DAT) allows the uptake of extracellular DA into presynaptic terminals, disrupting its function [1,2]. In a new model of rat, using biotechnologies, a stop codon was inserted into the structure of the SLC6A3 gene encoding for the DAT protein. In this way, DAT is still expressed but truncated at less than 70 aminoacids, resulting in an ineffective protein. In DAT-KO rats, the SLC6A3 gene is produced but functionally silenced.

Rats carrying both truncated DAT alleles (i.e., DAT-KO rats) are therefore in conditions of hyperdopaminergia, resulting in: hyperactivity, working memory deficits, and increased propensity to develop stereotypic behaviors [1,3,4]. In addition, KO rats exhibit circadian rhythm disturbances, in particular they typically exhibit difficulty in falling asleep. Therefore, these rats exhibit a typical behavioral profile, similar to that produced by a chronic treatment with psycho-stimulants [4].

Since they have only one functional allele for the DAT gene, heterozygous (DAT-HET) rats seem likely to be susceptible to epigenetic influences [2]. Although they exhibit a HET genotype, they may show different phenotypes based on the exclusive maternal or paternal derivation of the wild-type and mutated alleles. The maternal or paternal origin of the wild-type allele is called “asset” and its role is treated in another paper [5].(To conduct our recent studies on heterozygotes [6], two genealogical lines have been created, with known allelic derivation (one maternal and one paternal): 100% heterozygous (HET) offspring were obtained in both lines, by crossing a WT mother with a KO father (i.e., so-called “MAT rats”) versus by crossing a KO mother with a WT father (i.e., so-called “PAT rats”).

MAT-HET rats displayed some region-specific changes in DAT expression: compared to “classical” HET subjects, MAT-HETs displayed higher DAT density in dorsal striatum and prefrontal cortex [6,7]. As greater DAT availability may elevate threshold for dopamine action, impaired function was proposed within the cortico-striatal and cortico-thalamic circuits [6,7]. Hence, impaired updating of implicit memory with a deficit of top-down inhibitory control are likely, thus causing aberrant fear, social avoidance, and compulsive behavior (all observed in MAT-HET rats).

At this point, the first problem has arisen. The dams of the “paternal” line are KO, so they are in a hyperdopaminergic condition: as such, they normally tend to have no milk. For this reason, in order to investigate the PAT-HETs, we usually adopt this strategy: taking the whole MAT and PAT litters and inverting their dams at birth. So, the PAT offspring is cared by WT dams, while the other line has been called “K-MAT”, where the letter “K” indicates that these are MAT rats, adopted from and cared by KO dams (see Table 1).Significant evidence demonstrates that DA plays a predominant role in regulating the secretion of prolactin (PRL), a polypeptide hormone mainly synthesized and secreted by lactotrophic cells of the anterior pituitary. Extensive research in vitro and in vivo has shown that DA is a potent inhibitor of PRL release [8,9]. Through a direct effect on pituitary lactotrophs, DA inhibits the basally elevated secretory tone of these cells by binding to D2 receptors on their membranes [10]. The activation of D2 receptors causes a reduction of prolactin’s exocytosis, through a variety of intracellular mechanisms [11].

It has become evident, in some rare cases, that KO mothers may also have milk. Indeed, certain modulators of prolactin secretion can act at the hypothalamic level, inhibiting dopaminergic tone [11]. At the level of the pituitary gland, for example, estradiol functions as an anti-dopaminergic agent: it makes DA a less potent inhibitor of PRL secretion, perhaps through a reduction in the number of dopaminergic receptors expressed on the cell membrane of lactotrophs [12]. In addition to estrogen, other substances that show an anti-dopaminergic effect at the hypothalamic level are serotonin, GABA, and endogenous opioids [11].

Interestingly, the few KO dams who were able to breastfeed turned out to be maltreating towards their adopted MAT offspring. It is well known that the style of care experienced by females during childhood is able of shaping the style they will later adopt as mothers. However, at present these phenotypic effects are not entirely clear. We wondered if any maternal maltreatment, practiced by the KO grandmother on the MAT-HET future dam, could be inherited by the latter’s offspring in the form of epigenetic modulations. To investigate this issue, K-MAT rats caring for “MIK” offspring (the final “K” indicates the putative sequelae of *traumatic care*) were compared to a WT control group. The MAT-HET females, raised by KO dams (K-MAT epigenotype) and who became mothers in turn, were here found to be less inclined to provide adequate maternal care to their MIK offspring.

A reference group to assess MIKs would be MIXs, namely offspring of KO fathers and MAT dams (instead of K-MAT dams), whose circadian phenotype appears to be depressed [13]. In our study, instead, we will consider a slightly different epigenotype of rat, called “MUX” and obtained by taking a male MAT rat, bred with a female KO dam. Practically, compared to MIX rats, the two parents are inverted with each other. Their offspring were named MUX because of the rules detailed by Liberati et al. [2]:

(1) As for MIXs, the letter “M” shows the original “maternal” genealogy of the wild allele (coming from the father, and before that from the paternal grandmother);
(2) The letter “U” indicates fetal development in hyperdopaminergic uterus, because the natural dam is KO (here, there is the need of a postnatal adoption as well).

Rationale for this inversion comes from our recent findings [5], prompting us to conclude that the asset plays a more important role than maternal care; while the parental origin of the truncated DAT-KO allele had an important effect on the phenotype of the offspring, the type of maternal care had almost none [2]. The purpose was then to assess if reversing the asset (i.e., in the MUXs it is inverted versus the MIXs, due to subhealthy allele inherited from father) was also originating a depressive picture, possibly even worsened.

The present study is unique: maltreatment was generated in a spontaneous way from DAT-KO mothers to the offspring. Future studies may well address the separate contributions of genotype and upbringing factors. Ultimately, the present paper will serve a first step to evaluate both the trans-generational transmission of postnatal maternal impact and the epigenetic role of asset in DAT-heterozygous rats.

## 2. Methods

All rats were kept, according to an inverted light–dark cycle (lights off at 8 a.m. and on at 8 p.m.), in a room with controlled temperature and humidity (T 21 ± 1 °C; relative humidity 60 ± 10%) and with food (pellets Altromin-R, A. Rieper SpA, Vandoies, Italy) and water ad libitum. Behavioral data were analyzed by Repeated Measures ANOVA (RM-ANOVA), using StatView software (Abacus Concepts, Berkeley, CA, USA). The significance level was set at *p* ≤ 0.01, and post hoc analysis was performed using Tukey’s HSD test. All figures show the standard error of the mean.

### 2.1. Exp. 1

#### 2.1.1. Subjects

The experimental cohort of experiment 1 consisted of 21 female rats (12 WT and 9 K-MAT rats) and their postnatal lactating pups. These dams were either WT (themselves born to a WT mother and father) or heterozygotes who experienced altered maternal behavior (K-MAT: genetically offspring of KO male and WT female, with healthy allele from paternal grandmother, but with psychological trauma induced by the obsessive-compulsive care of the KO adoptive mother).

#### 2.1.2. Procedure

The analysis of maternal behavior was carried out Monday to Thursday for two weeks with observations divided into three time windows (10:30–11:00, 11:30–12:00 and 12:30–13:00). In each time window, each cage was observed for approximately one minute, before proceeding to the remaining cages: three readings (“shifts”) were produced every ten minutes for each cage. The behavior of the mother rat towards the pups was verified in the ethogram and coded with a thick in the corresponding checkbox. The maternal behaviors explored were: self-grooming, resting, eating, drinking, grooming pups, licking pups, moving pups by mouth, turning with tail in mouth, arcuate breastfeeding, prone breastfeeding, supine breastfeeding, covering pups with body, covering pups with sawdust, digging, and pups breastfeeding themselves when mother does other things.

The data collected by means of the ethogram were entered in the spreadsheet: numbers of thickswere then added together, per each of the three shifts and across the three time windows over the observation working days. A sum was thus obtained accounting for the totals of the three shifts, collected for individual dams (of course, divided into groups by their epigenotype). These data were entered into the StatView file for final analysis.

### 2.2. Exp. 2

#### 2.2.1. Subjects

The experimental cohort (64 adolescent male rats) consisted of the control WT group (offspring from WT mother and father), of MUX heterozygotes (offspring of KO female and MAT father adopted at birth from WT mothers) and of MIK subjects (genetically MIX rats but with K-MAT mother). These were the same offspring observed for experiment 1 above.

All day of delivery is defined as postnatal day (PND) zero. All pups were called at birth to 5 ± 1 males and 3 ± 1 females and were weaned at PND 22 ± 2; KO siblings of MIK and MUX pups were discarded.

#### 2.2.2. Procedure

The starting point of the experiment was forming of the sixteen yoked quadruplets, each with non-sibling pups of the same epigenotype; for each quadruplet formed at weaning (PND 22 ± 2), the male rats are marked with a blue felt tip, with one to three lines on their tail, while the female rat is left unmarked. Thus, rats were marked as “A” (female rat), ”B” (single-marked male rat), “C” (double-marked male rat) and “D” (triple-marked male rat). In the initial phase, in one cage with the female rat there was the male rat “B” with a single marking, while in the other yoked cage of the same quadruplet, there were “C” and “D” male rats with double and triple markings.

A 29-day protocol was adopted (start, PND 25; end, PND 55) in which there was an exchange (“swap”) of one of the males within each quadruplet that took place every three or four days. The male rats in the yoked cage were placed in the female rat’s cage, in a rotation schedule where all possible couples were formed to complete the cycle three times. The pairs are located in alternated and counterbalanced ways (in the sensor rack) according to epigenotype (WT, MIX and MIK), forming a matrix designed to avoid errors in the swap exchanges. Each row in the rack hosted yoked cages between columns one and three (WT rats) and between columns two and four (MIX or MIK rats).

On the day of the swap, fifteen minutes after the manipulation, systematic readings of one minute per cage were taken (same schedule as for maternal observations, see above), to ascertain whether the swap stimulated play behavior (data not shown).

#### 2.2.3. Apparatus

The continuous detection of locomotor activity proves to be an important tool to ascertain the conditions that the subject manifests in a given social frame. Several methods are used to evaluate this, such as the use of cameras [14], microwave radar [15], and systems capable of capturing the heat of the subjects [16,17].

In order to detect the peculiar characteristics of the sleep–wake cycle in the subjects, a system of sensors was used: each sensor was positioned above each cage at an angle of ±50° and anchored to a plate at the back of the cage. Each sensor was connected to a control unit that directs the readings to the computer via the parallel interface. Data collection was carried out via the Activiscope (Technosmart, Guidonia, Italy) software.

Raw data on locomotor activity were recorded 24/7 for approximately one month. In order to analyze the data, triplets from the 3 days immediately following a swap (i.e., the exchange of one male per pair) were first determined. Any fourth day on the same pair did not fit into the analysis and were eliminated. In order to group all the readings taken from the same pair of subjects, the three relevant triplets of days were identified and placed contiguously, resulting in nine adjacent days (consisting of three triplets of days) out of that pair of animals.

Finally, from these nine days, the average day (expressed by each pair, formed by the 24-hourly points) was calculated, always starting at 8 a.m. and ending at 7 a.m. on the following day. The average days calculated in this way formed a matrix of 48 rows (one for each pair) by 24 columns and were then entered into the StatView file for final analysis.

### 2.3. Analysis

For experiment 1, the ANOVA presents, a 2 × 2 factorial design, in which the “Between” factor represents the epigenotype of the mothers (two levels: WT vs. K-MAT) and the “Within” factor is divided into two levels (i.e., the 2 types, for attractor of interest and for the mode of lactation). In experiment 2, the ANOVA presents a 3 × 24 factorial design, where the “Between” factor (epigenotype) has 3 levels, which allow the WT control offspring to be compared with two groups of the HET genotype, derived from maternal lineage which was modulated either at a prenatal level (MUX: hyperdopaminergic uterine life) or at postnatal level (MIK: psychologically traumatic childhood as a result of maternal K-MAT care). Finally, the “Within” factor represents the 24 hourly points of the average day.

Behavioral data were analyzed by Repeated Measures ANOVA (RM-ANOVA). The significance level was set at *p* ≤ 0.05 for maternal observations and at *p* ≤ 0.01 for circadian cycles; post hoc analysis was performed using Tukey’s test.

## 3. Results

### 3.1. Maternal Behavior

**Figure 1:**
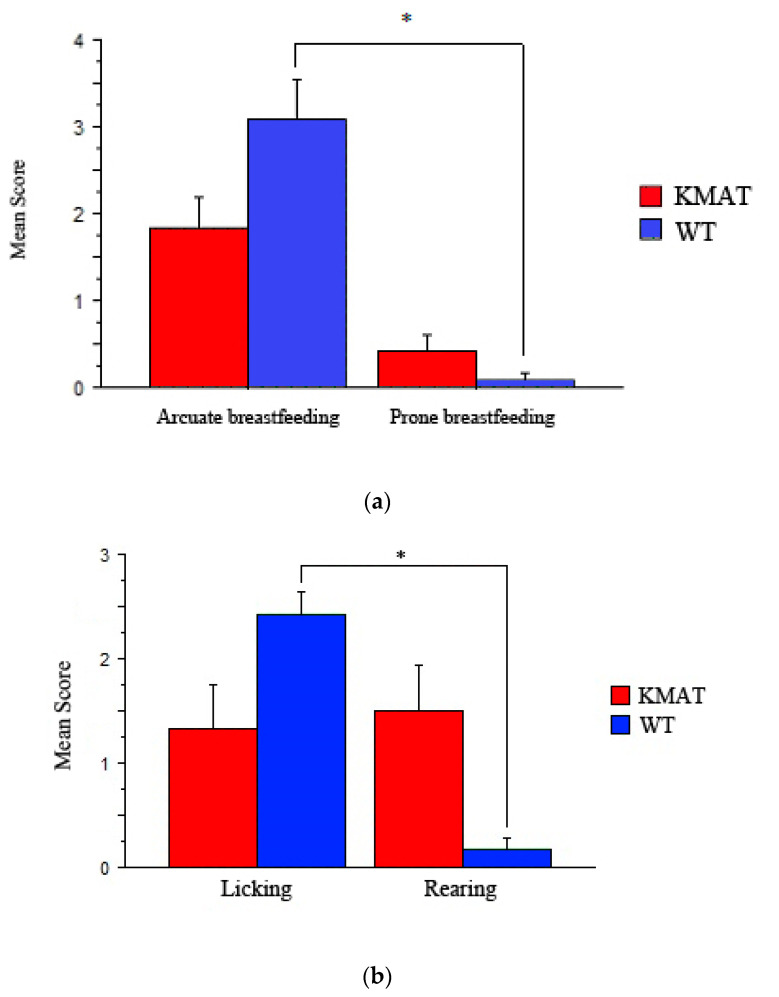
(**a**) Patterns of breastfeeding: formal comparison of arched and prone mode, expressed by mothers of WT and K-MAT epigenotype (n = 12 WT; n = 9 K-MAT). K-MAT rats are MATs (heterozygous offspring of WT dam and KO father) that have been fostered to (and abused by) KO mothers (hence the initial “K”). The asterisk (*) indicates the statistically significant difference between either breastfeeding mode in WTs. The observations of maternal behavior covered a two-week period between postnatal day (PND) 2 and 16. (**b**) Attractors of interest: formal comparison on attention directed towards offspring (licking) or outwards (rearing), expressed in dams of WT and K-MAT epigenotype (n = 12 WT; n = 9 K-MAT). Observations carried out using the ethogram and the methods described in Methods; dams are the same as above (**a**). The asterisk (*) shows the statistically significant difference between the two attractors of interest in the WT mother. As expected, the latter is preferring to take care of her offspring (i.e., licking them) rather than expressing any interest for external attractors. The K-MAT mother, on the contrary, is much more attracted by external environment rather than by caring for her offspring.

We compared the differential effect of the two epigenotypes (WT vs. K-MAT) on the type of lactation (i.e., Arcuate vs. Prone). There is a significant interaction (F_1,22_ = 7.053; *p* = 0.144) between the type of lactation and epigenetics of the mother: a statistically significant effect was found in WT dams but not K-MAT ones. With regard to WT dams, we observed (Figure 1) how breastfeeding occurs in a more active mode, compared to a passive one (Tukey GF = 22; K = 2; threshold = 2.312). In contrast, in the case of K-MAT dams, there is a less pronounced and non significant difference between the two breastfeeding modes. 

**Figure 2:**
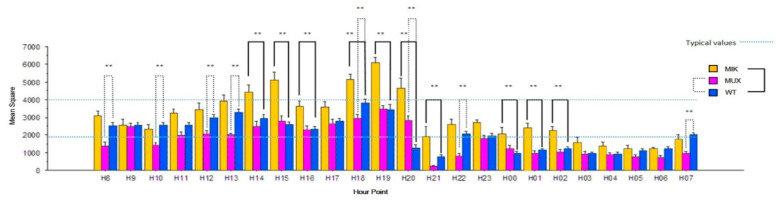
Daily average of locomotor activity in hourly counts for various rat groups: MIK (heterozygous offspring of K-MAT mothers and KO fathers), MUX (heterozygous offspring of KO mothers and MAT fathers, fostered to WT dams) and the WT control group. MIK are genetically MIX (yet with K-MAT rather than MAT mother). The infrared sensor system allowed the measurement of the home-cage locomotor activity: the circadian cycle was monitored over a period of one month, from PND 25 to 55; the daily average was subsequently calculated. The asterisks (**) relate to statistically significant differences (*p* < 0.01) between either MIK or MUX and WT (see plain or dashed connectors, respectively).

We compared the differential effect of the two epigenotypes (WT vs. K-MAT) on the type of attractor: in other words, the analysis compared whether the interest of the dam was directed towards the offspring (via expression of “Licking”) or rather directed outwards (via expression of “Rearing”). There is significant interaction (F_1,22_ = 12.866; *p* = 0.0016) between the attractor type and maternal epigenetics. Relative to WT dams, pups occur significantly (Figure 2) as attractors of interest (Tukey GF = 1.22; K = 2; Threshold = 0.983). In the case of K-MAT dams, however, there is non-significant difference between the two sources of attraction, as the two levels are strangely comparable to each other. Activities, in other words, are equally directed towards offspring for maternal care and towards the environment with an outward directed rearing.

### 3.2. Circadian Cycles

The experimental cohort (64 rats) consisted of the control group WT (36 offsprings from mother and father both WT); and 2 groups of second-generation heterozygotes, the MIK (12 rats) who are MIX but with a K-MAT mother (ergo, encounter of contaminated\healthy allele from uterus of natural maternal grandmother, but also *traumatic care* from adoptive maternal grandmother), and MUX (16 rats) that is heterozygous offspring from a female KO and a male MAT, (ergo, encounter of contaminated\healthy allele from uterus of paternal grandmother).

The ANOVA presents a factorial design in which the between factor “epigenotype” has three levels: the effect over the offspring of the KO genotype in the maternal pedigree compares to the control WT in two ways: either (1) with the postnatal level of the adoptive KO grandmother (generating the traumatic infancy and altered postnatal care of their own K-MAT mothers) or (2) with the prenatal level of directly KO-damgestation (in terms of their own hyperdopaminergic uterine life). Then, there is the within “time” factor, with 24 repeated-measure levels.

We compared the differential effect on the 24-h cycle among the MIK, MUX and WT epigenotypes (Figure 1). There was a significant interaction between epigenotype and time of day (F_46,1380_ = 7.419; *p* =< 0.0001). The MIK epigenotype, despite being a second-generation heterozygote with a healthy allele from the maternal line, produces hyper locomotor activity during wakefulness, well above typical values normally ranging between 2000 and 4000 (Tukey GF = 1380; K = 10; Threshold = 860.251). Furthermore, the MIK epigenotype appears to have locomotor peaks at mid of cycle (hour 15:00) and especially before the lights are turned on (hour 18:00–hour 21:00).

Compared to the MUX and WT genotypes, MIK describes an abnormal rebound of activity and wakefulness even in resting hours, which extend until 4 a.m. The sleep state is observed for MIK rats during only two hours, in marked contrast to the other two genotypes.

At hour 20:00 and hour 22:00, there is a pronounced depressive profile of MUX compared to WT genotypes, with the MUX being less active than latter and this happens in the whole pre change phase. At hour 21:00 and during the depressive-like dissonance of the MUX genotype is less pronounced and not significant compared to WT.

## 4. Discussion

Environmentally-derived epigenetic changes are known as: (1) intergenerational, if present only between two contiguous generations, and (2) transgenerational, if translatable into subsequent generations even in the absence of the environmental component that originally determined the behavioral change. The study by [18] found that the administration of the fungicide vinclozin in pregnant female rats caused spermatogenic apoptosis in the offspring from the first to the fourth generations. The study by Dias and Ressler [19] found that olfactory conditioning carried out in mice develops transgenerational inheritance at the epigenetic, behavioral and neuroanatomical levels. Transmission of the behavioral phenotype to the offspring conveys information acquired epigenetically, both prenatally and postnatally through direct mother/offspring contact, by acting on the developing gonads in the fetus. The consequences on the behavioral phenotype that occur in transgenerational ways were presently determined by reconstructing the inheritance pathway of both healthy and mutated alleles.

The genealogy of our experimental subjects exploits the use of DAT KO rats as one of the two parents: they manifest significantly hyperactive and stereotyped behavior characterized by restless exploration of the environment [3] and reduced liability to fall asleep [20]. In the present study, we focused on the offspring of specific DAT-HET epigenotypes, namely using either MAT rats as fathers or K-MAT rats as mothers: their offspring are labeled, respectively, MUX and MIK [2]. In our experimental group, there were MAT females who had a psychologically traumatic experience during childhood, having been abused by their KO adoptive mother. In a first control group, heterozygous MAT males (with a maternal healthy allele) became fathers and carried this wild-but-somewhat-altered allele through the spermatozoon into a KO female, giving birth to MUX: these are heterozygous offspring with such a sub-healthy allele coming from the paternal grandmother. One of their peculiarities is that fetal development took place in a hyper-dopaminergic uterus.

There were significant differences between the MUX and MIK epigenotypes: when compared to WT control rats, while the latter showed hyperactivity, the former showed a behavioral depressive profile with a moderate decrease in circadian locomotor activity.

In the literature, an indistinct classification of heterozygous rat offspring is usually employed. Thus, a clear distinction between maternal (M) and paternal (P) assets in the offspring is missing [2]. However, our preliminary data [5] show that asset plays an important role in the behavioral manifestations of rats: the locomotor and social phenotype related to the P asset results to be slightly more severe than the M one, suggesting a parental-origin effect [21,22]. The prenatal phase is essential for these epigenetic changes [23].

We proposed recently that when “virgin” (that is, of pure WT lineage) alleles encounter for the first time KO alleles, an intra-allelic epigenetic process may occur within heterozygous MAT zygotes [13]. However, this type of alteration seems to affect the offspring of MAT rats at the first generation only. In successive generations, this epigenetic alteration is no longer having observable effects on the phenotype.

### 4.1. Evidence of Transgenerational Transmission

Present experimental subjects are called K-MAT, where “K” recalls the KO mother and her cares: such a dam licked and moved the pups continuously and compulsively, because of the DAT-related obsessive-compulsive behavioral profile. According to the rules established in [2], MIX pups belong to the second heterozygous generation with a sub-healthy allele from the maternal grandmother (i.e., they come from a heterozygous MAT mother); MIK are MIX with a K-MAT rather than MAT mother. Our aim was to evaluate the sequelae on MIK due to the maternal behavior of K-MAT, possibly altered by KO grand-dams. The MUX, descending as mentioned above from a KO mother, manifested a much less active behavioral rhythm, possibly due to deficits in the social-interaction profile. We hypothesize that this kind of sequelae depends on having inherited alterations at the epigenetic level from the MAT father; in particular, we propose that a sub-healthy allele carries the signs of having met a KO allele. This is in agreement with the study by Perera and Herbstman [23], which points out that prenatal exposures are fundamental. The genetic imprinting is determined during germ cell development, and it alters individual characteristics over the whole life cycle. The sperm of a MAT male would hence carry a sub-healthy allele, compared to alleles transmitted by a WT male rat.

The theory posits that environmental and social inputs result in epigenetic changes that allow offspring to adapt rapidly to possibly complex contexts. The epigenome could be transmitted to offspring through DNA methylation and through sperm [24,25]. In accordance with this theory [26], epigenetic change impacts the reproductive haploid cell: during stressogenic events, the extracellular vesicles of the epithelial cells in the epididymis alter the sncRNA content in the spermatozoa as they pass through [27,28,29]. Life experiences in infancy, whether normal or traumatic, contribute to influencing the next generation via microRNA [30,31]: accordingly, a microRNA direct delivery in embryos results in behavioral changes both in target animals and their offspring, even in the absence of any traumatic event [31]. This results in behavioral and physiological changes in the offspring. In our MAT rats, where the healthy allele met the truncated allele for the first time, spermatogenesis could be affected similarly.

The MIK offspring had a double source of alterations in their phenotype: the maternal inheritance line conferred the sub-healthy allele, but in addition they received maternal care from their K-MAT mother, who was found to be extremely neglectful. The K-MAT dams, when pups, received a psychological trauma during childhood from their own KO mother: such sequelae in turn were passed on to MIK offspring. The activity shown by the MIK rats significantly exceed the normal range, especially before the lights at the facility are switched on (hour 21:00), including extended wakefulness during the lit phase of the cycle. We thus hypothesize that transmission shifts from the genetic to the epigenetic domain, as the environmental trauma suffered by K-MATs is passed on to the next generation. Indeed, maternal behavior triggers epigenetic and hormonal responses in the offspring, informing the plasticity of genetic expression, based on the social and environmental context.

The act of licking the body and/or genitals has been shown to be an important discriminator in offspring growth: mothers with high levels of arch-back lactation allow their offspring (once adult) to face new situations with reduced behavioral stress response and phobia, compared to offspring receiving low levels of such care during lactation [32]. The optimal care they have taken in the postnatal period reduces the HPA activation during stress, persisting throughout life [33,34,35,36]. In rats reared with low levels of licking by their mothers, conversely, DNA methylation on the oxytocin gene is present [37]. Maltreatment in the postnatal phase induced risk factors for the development of a depressive profile, by acting on the polymorphic region associated with the serotonin transporter [38].

Our rat model was devised without the need to induce an exogenous trauma thanks to the opportunity of having a mother who was herself traumatized in childhood. The literature on trans-generational sequelae following stress on rats is still lacking models aimed at reproducing forms of maternal maltreatment in an ecological mode. Our study, the first of its kind, differs from laboratory models of exogenously induced stress and trauma: it exploits the impact of DAT-KO mothers on K-MAT offspring and subsequent impact on their own MIK offspring. This grand-maternal influence developed, in the female K-MAT rat which later became a mother, a negative behavior towards the offspring. This is thus a realistic model, mimicking situations existing in real life, thus maintaining a high etho-ecological profile.

### 4.2. Remarks on Maternal Cares

In the present paper, it is shown how changes in maternal K-MAT behavior, due to the own KO mother’s, can result in a transgenerational change in MIK offspring. The present pilot study deserves future investigation, as we are presently not able to separate two combined aspects accounting for the effects observed on K-MAT mothers (namely, to dissect the role of abnormal maternal care, had from a KO mother, from the role of vulnerable genotype). To do so, a group of WT pups cared by a KO dam (namely, an additional K-WT group) would be needed; in turn, a non-maltreated yet vulnerable genotype (i.e., MUX) presently controls for the simple effect of genotype.

Among mammals, it has been suggested by Meaney J. [33] that natural selection may have shaped offspring to respond to slight variations in parental behavior, in order to predict what environmental conditions they will face once they become independent of them. Numerous studies in the literature show that, in all species, stressed parents expose their offspring to adverse consequences [39,40].

Several results suggest that maternal stressful experience is translated into epigenetic mechanisms, through molecules which are inherited by the future offspring [41,42,43]. Parents exposed to stress may also confer vulnerability to their offspring through behavioral alterations that change their ability to be good caregivers. One final question concerns the precise mechanisms by which these maternal effects are transmitted. The process whereby stress experienced by the parent has lasting consequences on the functioning of future offspring is called “intergenerational transmission”. In non-human primates and rodents, inadequate maternal care predicts an atypical emotional development in the offspring, in particular, higher levels of anxiety, and also influences the type of maternal care that the female offspring will provide once they become adults [44,45,46,47].

The intergenerational transmission of maternal effects has been extensively studied in the rat. Different studies show that the early care style that female rats receive from their dams is a reliable predictor of the type of care that they will adopt when they become dams [48,49,50,51,52]. In rats, maternal caregiving behaviors such as retrieving pups from the nest, licking and grooming, provide pups a tactile stimulation, and are associated with offspring’s reduced stress responses in adulthood [53,54]. In several lines of research [44,55] female offspring who had early experiences with dams, who licked and groomed a lot, showed higher levels of licking when adult.

Other studies on isolation from maternal contact [56,57] found that such early experiences did not completely interrupt the female rat’s future ability to engage in species-specific maternal behaviors but did impair the frequency of these behaviors. Rat females raised without maternal contact, once they became dams, spent significantly less time engaging in the caring behaviurs than the control group [48,49,58,59,60,61,62]. Deficits in maternal behavior, and in particular, the deficit in licking the pups, is thus transferred to the next generation [48].

### 4.3. Translational Relevance and Limitations

Childhood trauma is a precursor for a variety of psychological disorders, such as anxiety, mood instability and personality changes [63]. Childhood maltreatment also increases the possibility of developing overt mental disorders in adulthood [64] with a particularly high incidence of obsessive compulsive disorder [63,65]. It can be hypothesized that parents who mistreat their children transfer this behavioral pattern to them, in that they will tend to repeat what they have suffered when they become adults. Children who have been mistreated by their parents are more likely to replicate the same in the next generation [66,67]. Such intergenerational transmission could be caused by the combination of social and genetic factors [68] so that the two are closely intertwined. Children with obsessive-compulsive mothers are six times more likely to develop OCD in adulthood than those without [69]. According to Siegel [70], children who are genetically vulnerable and have been traumatized by their genetically vulnerable parents may, as adults, just replicate what they have learned. Mothers with OCD [71] are classified as depressed and less sensitive in interactions with their children. In the literature, it is highlighted that [72] the poor empathic relationship in OCD mothers is a predictor of postnatal depression [73]. At the same time, genetic heritability of depression has been estimated to be 40% [74]. To dissect the separate roles of poor empathy vs. genetic OCD is practically impossible. This is why we did not use a group of WT pups cared by the KO mother rats. This is one major limitation of this study.

In adults who have suffered childhood trauma, resilience or vulnerability to psychopathology is characterized by a clear-cut sexual dimorphism, interweaving with biological factors. It has been observed how childhood trauma is associated with poorer social cognition in both genders but more depressive symptoms in women [75]. In contrast to previous studies, Pruessner et al. [76] indicate how childhood trauma led to a more significant psychosis in males. This contrasting evidence makes it difficult to take a conclusive statement, so that more studies may be warranted for the determination of sexual diversity in depressive disorders. Indeed, sexual dimorphisms may widen the likelihood that women suffer a major depressive disorder [77,78] to an extent about twice that of men.

### 4.4. Conclusions

The dopaminergic dysregulation is a key feature in social deficits [79]. Heterozygous (DAT-HET) rats show antisocial tendencies [3], because a reduction in reward processing can decrease the prosocial propensity [80] and consequently result in an exaggerated fear of danger [7]. From our previous results, we concluded that offspring of MAT females do not interact regularly and efficiently with each other nor with other WT rats [13]. Social skills are further impaired in the offspring of MAT males (i.e., the current MUXs) asthe (sub)healthy allele has probably been epigenetically shaped across generations [81].

A dysfunctional dopaminergic system, as happens in DAT-HET rats, also results in disturbances of sleep, which cause important changes in circadian rhythm [82,83,84]. The anomalies in the resting phase of MIKs (see Section 3.2) could have well-defined effects in a translational perspective: any disruption of circadian rhythms can lead to a variety of psychological and mental problems [20]. According to Tibbitts [85], one in five adults suffers from insomnia; inter-generational transmission of such problems warrants investigation.Further studies are needed to explore the full consequences of the circadian alterations in rats of MIK epigenotype. Current planning foresees to breed MIK females with WT males to obtain a SIK offspring. Therefore, identifying risk factors resulting in the epigenetic emergence of these circadian diseases is critical.

## Figures and Tables

**Table 1:** Epigenotypes: abbreviations’ legend.

Abbreviation	Explanation
WT = wild-type	Most frequent genotype, for dopamine transporter (DAT), in the natural population
KO = knock-out	Homozygote knocked-out for the DAT gene, is usually obtained as sibling of other epi-genotypes
HET = heterozygous	Inheriting one healthy and one knocked-out allele from each parent
MAT = maternal	Heterozygous specimens, offspring of a KO male and a WT female
PAT = paternal	Heterozygous specimens, offspring of a WT male and a KO female
K-MAT (see text)	MAT rats, but adopted and cared (maltreated) from KO dams
MIX = mixed with KO siblings	Heterozygous offspring of a MAT female and a KO male
MUX = mixed, and born from KO uterus	Heterozygous offspring of a KO female and a MAT male
MYX = mixed, and its dam in KO uterus	Offspring from KO male and MUX female, that is in turn heterozygous offspring from a KO female and a MAT male (i.e., the maternal grandmother was KO and dam was born from KO uterus)

## Data Availability

All original data leading to this paper are stored on a computer located at ISS in the office of the corresponding author. All raw data can be made available upon request.
